# Elavl3 is essential for the maintenance of Purkinje neuron axons

**DOI:** 10.1038/s41598-018-21130-5

**Published:** 2018-02-09

**Authors:** Yuki Ogawa, Kyoko Kakumoto, Tetsu Yoshida, Ken-ichiro Kuwako, Taisuke Miyazaki, Junji Yamaguchi, Ayumu Konno, Junichi Hata, Yasuo Uchiyama, Hirokazu Hirai, Masahiko Watanabe, Robert B. Darnell, Hideyuki Okano, Hirotaka James Okano

**Affiliations:** 1 Division of Regenerative Medicine, The Jikei University School of Medicine, 3-25-8 Nishi-Shimbashi, Minato-ku, Tokyo, 105–8461 Japan; 2 Department of Physiology, Keio University School of Medicine, 35 Shinanomachi, Shinjuku-ku, Tokyo, 160–8582 Japan; 3 Laboratory for Marmoset Neural Architecture, Brain Science Institute RIKEN, 2-1 Hirosawa, Wako, Saitama, 351–0198 Japan; 4 Department of Anatomy, Hokkaido University Graduate School of Medicine, Kita 15, Nishi 7, Kita-ku, Sapporo, 060–8638 Japan; 5 Department of Cellular and Molecular Neuropathology, Juntendo University Graduate School of Medicine, 2-1-1 Hongo, Bunkyo-ku, Tokyo, 113–8421 Japan; 6 Department of Neurophysiology & Neural Repair, Gunma University Graduate School of Medicine, 3-39-22 Showa-machi, Maebashi, Gunma, 371–8511 Japan; 7 Research Program for Neural Signaling, Division of Endocrinology, Metabolism and Signal research, Gunma University Initiative for Advanced Research, 3-39-22 Showa-machi, Maebashi, Gunma, 371–8511 Japan; 8 Laboratory of Molecular Neuro-Oncology and Howard Hughes Medical Institute, The Rockefeller University, 1230 York Avenue, New York, NY 10065 USA; 9 Present Address: Immunoregulation for the treatment of inflammation-related disorders, IBRI Laboratory, Foundation for Biomedical Research and Innovation, 2-2 Minatojima-minamimachi Chuo-ku, Kobe, 650-0047 Japan

## Abstract

Neuronal Elav-like (nElavl or neuronal Hu) proteins are RNA-binding proteins that regulate RNA stability and alternative splicing, which are associated with axonal and synaptic structures. nElavl proteins promote the differentiation and maturation of neurons via their regulation of RNA. The functions of nElavl in mature neurons are not fully understood, although Elavl3 is highly expressed in the adult brain. Furthermore, possible associations between nElavl genes and several neurodegenerative diseases have been reported. We investigated the relationship between nElavl functions and neuronal degeneration using *Elavl3*^−/−^ mice. *Elavl3*^−/−^ mice exhibited slowly progressive motor deficits leading to severe cerebellar ataxia, and axons of *Elavl3*^−/−^ Purkinje cells were swollen (spheroid formation), followed by the disruption of synaptic formation of axonal terminals. Deficit in axonal transport and abnormalities in neuronal polarity was observed in *Elavl3*^−/−^ Purkinje cells. These results suggest that nElavl proteins are crucial for the maintenance of axonal homeostasis in mature neurons. Moreover, *Elavl3*^−/−^ mice are unique animal models that constantly develop slowly progressive axonal degeneration. Therefore, studies of *Elavl3*^−/−^ mice will provide new insight regarding axonal degenerative processes.

## Introduction

Elav-like (Elavl or Hu) proteins are the mammalian homologs of the *Drosophila* Elav protein. Elavl proteins were discovered as antigens from patients with paraneoplastic neurological syndrome accompanied by small-cell lung cancer^1^. Thereafter, four Elavl family members - Elavl2 (Hel-N1, HuB), Elavl3 (Ple-21, HuC), Elavl4^2–5^ (HuD), and Elavl1^6,7^ (HuA, HuR) - have been identified. Elavl2, Elavl3, and Elavl4 are also known as neuronal Elavl (nElavl) because these three Elavl proteins are specifically expressed in peripheral and central neurons throughout development. However, Elavl1 is ubiquitously expressed in many types of cells^7^.

Elavl proteins are RNA-binding proteins that directly interact with target RNAs. They regulate alternative splicing of immature RNAs (hnRNAs) in the nucleus^8–11^ and regulate the stability of mature RNAs (mRNAs) in the cytosol^12–16^. Target RNA recognition is based on their primary sequences, such that nElavl proteins mainly target RNAs, which have U-rich elements with interspersed purine residues in introns or untranslated regions. Our previous study identified more than 100 target RNAs of nElavl proteins using a HITS-CLIP assay^17^, a method for identifying target RNAs of RNA-binding proteins that incorporates UV cross-linking, immunoprecipitation, and high-throughput sequencing. Subsequent gene ontology analysis revealed that many of these targets function in the maintenance of axonal and synaptic structures. nElavl proteins are known to promote neuronal differentiation and maturation through these regulatory functions on RNA^11,18–20^.

nElavl proteins have also been implicated in neurodegenerative diseases such as Alzheimer’s disease^21,22^ and Parkinson’s disease^23–25^. Furthermore, a recent study demonstrated that the function of nElavl proteins is suppressed in Alzheimer’s disease brains^26^. We and others have studied how the suppression of nElavl proteins affects the brain using Elavl4-knockout (*Elavl4*^−/−^) mice^19,27^. *Elavl4*^−/−^ mice exhibited dendritic morphology deficits in cortical neurons and behavioral abnormalities, but did not exhibit neurodegenerative features. Although some mutations of Elav is lethal to embryonic *Drosophila*^28^, the effect of Elavl4 depletion in *Elavl4*^−/−^ mice was not as drastic as expected.

We hypothesized that these results are explained by the functional compensation by Elavl3 and/or Elavl2. Because the majority of neurons constantly express several types of nElavl, these neurons still express nElavl proteins in *Elavl4*^−/−^ mice. Double knockout (DKO) of Elavl3 and Elavl4 dramatically reduces total nElavl proteins in the brain. However, the DKO is lethal to mice several hours after birth^17^, and therefore, does not provide a suitable model to study the relationship between chronic human brain diseases and the function of nElavl proteins.

To examine the function of nElavl proteins in the adult brain, we focused on a specific type of neuron. In our previous study, we showed that most neurons expressed Elavl3 together with Elavl4 and/or Elavl2, with the exception of cerebellar Purkinje cells and hippocampal granule cells, which only express Elavl3. These two types of neurons become completely null for nElavl proteins in *Elavl3*^−/−^ mice^17^, although other neurons still express nElavl proteins. *Elavl3*^−/−^ mice were born healthy, viable, and had a normal life span, but exhibited slowly progressive motor deficits leading to severe cerebellar ataxia. Axons of *Elavl3*^−/−^ Purkinje cells were swollen (spheroid formation), and synaptic formation was disrupted at cerebellar nuclei. In this study, we assessed how the axons of nElavl-null Purkinje cells degenerate and *Elavl3*^−/−^ mice exhibit cerebellar ataxia.

## Results

### *Elavl3*^**−**/**−**^ mice exhibit cerebellar degeneration

**Figure 1:**
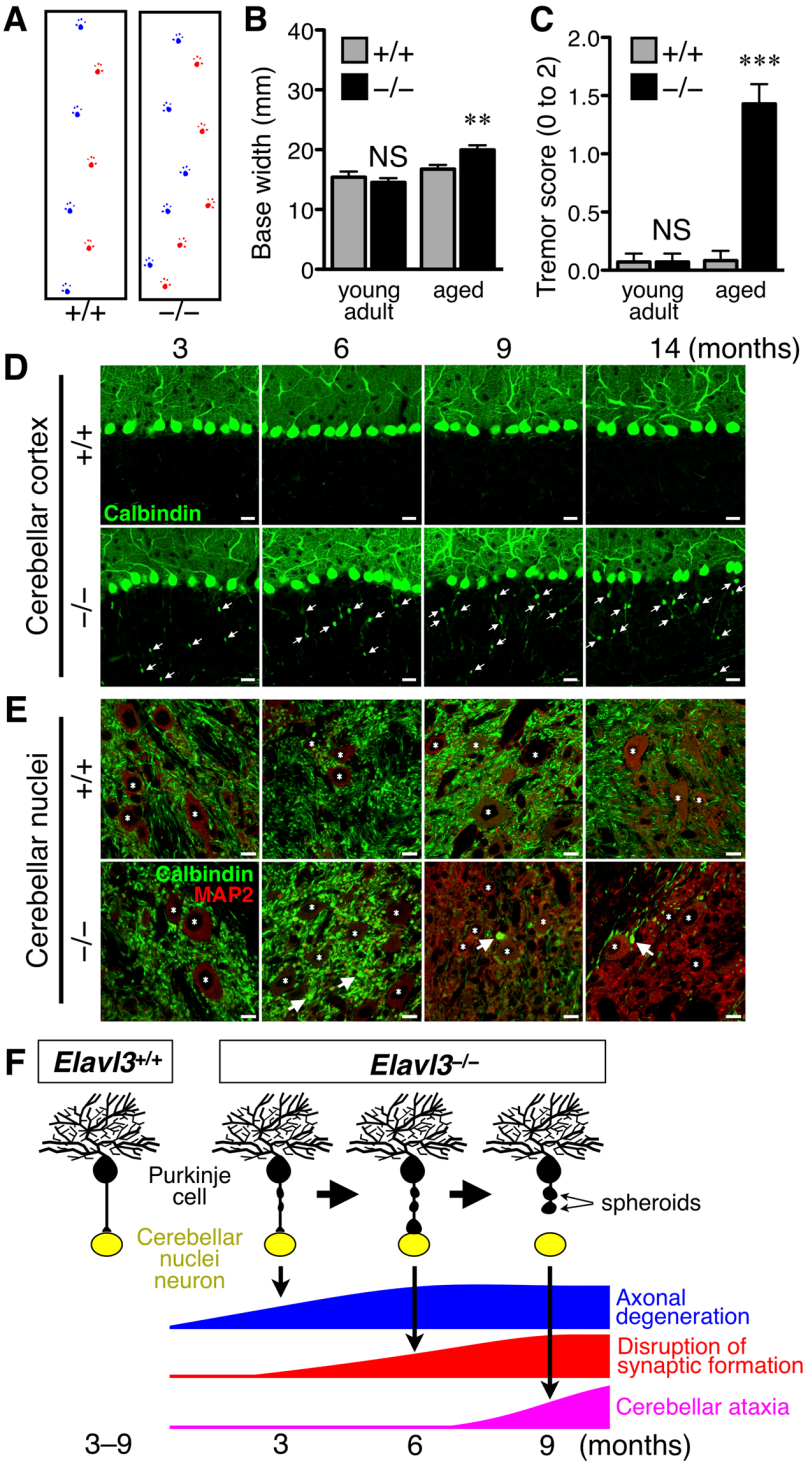
Behavioral and morphological analysis of *Elavl3*^−/−^ mice. (**A**–**C**) Behavioral analysis of *Elavl3*^−/−^ mice. (**A**) Representative forelimb footprints of 7-months-old *Elavl3*^+/+^ and *Elavl3*^−/−^ mice. (**B**) Aged (6–7 months old) *Elavl3*^−/−^ mice exhibited significantly wider forelimb base width compared to *Elavl3*^+/+^ mice (n = 7, 7, 6, and 7 mice for each group). (**C**) Aged *Elavl3*^−/−^ mice exhibited tremor during walking (n = 7, 7, 6, and 7 mice for each group). These phenotypes were not detected in young adult (2–3 months old) mice. **P < 0.01, ***P < 0.001, NS, not significant (Student’s t-test). (**D** and **E**) The cerebellar sections of *Elavl3*^+/+^ and *Elavl3*^−/−^ mice at 3, 6, 9, and 14 months of age were immunostained for calbindin and MAP2. (**D**) Axons of *Elavl3*^−/−^ Purkinje cells were swollen (spheroid formation) in the granule cell layer. Arrows indicate spheroids. Scale bar, 20 μm. (**E**) Axonal terminals of *Elavl3*^−/−^ Purkinje cells were swollen and synaptic formation was disrupted in the cerebellar nuclei with age. The density of axonal terminals of Purkinje cells at the cerebellar nuclei is quantified in Supplementary Fig. S2F and G. Arrows and asterisks indicate swollen axonal terminals and somata of cerebellar nuclei neurons, respectively. Scale bar, 10 μm. (**F**) Schematic model showing the degenerative processes of axons of *Elavl3*^−/−^ Purkinje cells.

In a previous study^17^, young adult *Elavl3*^−/−^ mice were shown to exhibit motor deficits in the rotarod test and seizures in electroencephalography analysis. In fact, young adult *Elavl3*^−/−^ mice exhibited abnormal step cycle in our gait test (Supplementary Fig. S1). Furthermore, in the present study, we found that the gait abnormalities of *Elavl3*^−/−^ mice became more prominent with age (>7 months old) as represented in the forelimb base width (Fig. 1A,B, and Supplementary Fig. S1). Aged *Elavl3*^−/−^ mice also exhibited apparent tremor (Fig. 1C and Supplementary Movie S1) and postural reflex impairment (Supplementary Movie S2). Because cerebellar Purkinje cells are null for nElavl proteins in *Elavl3*^−/−^ mice^17^, we focused our analysis on cerebellum and Purkinje cells as the cause of motor deficits. The cerebella of *Elavl3*^−/−^ mice appeared slightly smaller compared to *Elavl3*^+/+^ mice at all ages, but the gross anatomy of the cerebellum was not markedly different between *Elavl3*^+/+^ and *Elavl3*^−/−^ mice (Supplementary Fig. S2A–E). In addition, no apparent changes were observed in the anatomical architecture of the *Elavl3*^−/−^ cerebellum with aging (Supplementary Fig. S2A). *In vivo* magnetic resonance imaging (MRI) analysis revealed that the cerebellar volume of *Elavl3*^−/−^ mice reduced approximately to 88% compared to *Elavl3*^+/+^ mice at 8 months of age, and the volume of whole brain without cerebellum was not significantly different (Supplementary Fig. S2B). Immunohistochemical analysis also revealed that the thickness of both molecular layer and granule cell layer in the cerebellum of *Elavl3*^−/−^ mice was thinner compared to *Elavl3*^+/+^ mice (Supplementary Fig. S2D and E). Remarkably, pathological features of degeneration were observed specifically in the axons of Purkinje cells. These axons were swollen (spheroid formation) in the granule cell layer in animals as soon as 3 months of age (Fig. 1D). These spheroids were observed in all lobes of both vermis and hemisphere (data not shown). Moreover, axonal terminals of Purkinje cells were also swollen and gradually decreased at all areas of cerebellar nuclei in animals older than 6 months of age (Fig. 1E and Supplementary Fig. S2F and G). These data indicate that axonal degeneration develops age dependent manner. In addition, *Elavl4*^−/−^ mice^19^ did not exhibit such degeneration of the axons of Purkinje cells (data not shown) because Purkinje cells express only Elavl3 protein among nElavl proteins^7^.

Although axons were markedly degenerated in *Elavl3*^−/−^ mice, the somata and dendrites remained largely intact. The laminar structures of the cerebellum and some synaptic formation of Purkinje cells with climbing fibers and parallel fibers were retained even in the animals older than 24 months of age (Supplementary Fig. S3). We observed that the number of VGLUT2 positive puncta in *Elavl3*^−/−^ mice decreased approximately to 60%; however, the reason of the reduction is not clarified yet.

The time course of axonal degeneration and ataxia symptoms observed in *Elavl3*^−/−^ mice is summarized in Fig. 1F. Briefly, at 3 months of age, gross anatomy of cerebella and laminar structure of cerebellar cortexes looked normal. Although apparent spheroids appeared within the axons, Purkinje cells projected to the cerebellar nuclei. Axonal degeneration gradually proceeded, followed by the swelling of the axonal terminals and disruption of synaptic formation in the cerebellar nuclei by 6 months of age. Severe axonal degeneration finally prevented the retention of sufficient neuronal circuits in the cerebellum by 7 months of age, causing cerebellar ataxia in *Elavl3*^−/−^ mice.

### Histological analysis of spheroids

**Figure 2:**
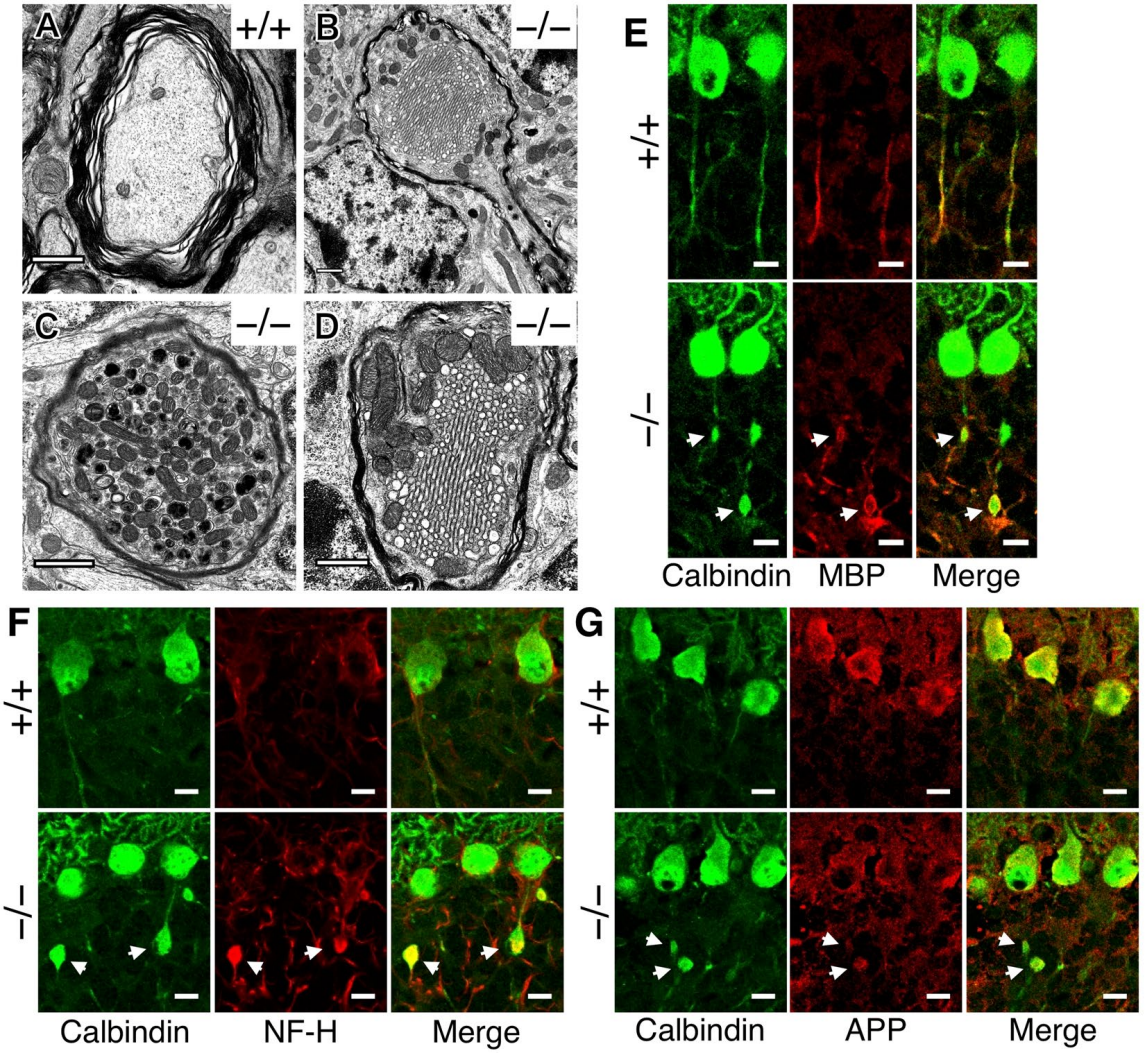
Histological analysis of spheroids. (**A**–**D**) Ultrastructures of cerebellum of 5- to 9-months-old mice were analyzed via transmission electron microscopy. (**A**) A representative image of normal axons of *Elavl3*^+/+^ Purkinje cells. (**B–D**) Representative images of axonal spheroids detected in *Elavl3*^−/−^ Purkinje cell. (**B**) The boundary that just enters from the axon to the spheroid. (**C** and **D**) Subcellular organelles such as mitochondria (**C** and **D**), multiple membranous bodies (**C**), and smooth endoplasmic reticulum (**D**) are observed in spheroids. Note that spheroids are surrounded by myelin sheaths. Scale bar, 1 μm. (**E–G**) The cerebellar sections of *Elavl3*^+/+^ and *Elavl3*^−/−^ mice at 9 months of age were immunostained with the indicated antibodies. Spheroids were surrounded by myelin sheaths (**E**) and filled with neurofilament-H (NF-H; **F**) and amyloid precursor protein (APP; **G**). Scale bar, 10 μm.

We analyzed the shape and content of spheroids via electron microscopy. Axons of Purkinje cells of *Elavl3*^+/+^ mice were surrounded by myelin sheaths, and their mean diameter was approximately 1 μm or less (Fig. 2A). However, axons of *Elavl3*^−/−^ Purkinje cells exhibited various abnormalities. Although these axons were also surrounded by myelin sheaths (Fig. 2B–E), their axonal diameter was enlarged at spheroids (Fig. 2B). Some spheroids were packed with subcellular organelles, such as mitochondria and multiple membranous bodies (Fig. 2C) and smooth endoplasmic reticulum (Fig. 2D).

We further examined whether proteins were also accumulated with subcellular organelles in spheroids. Immunohistochemical analysis revealed the accumulation of neurofilament-H (NF-H; Fig. 2F) and amyloid precursor protein (APP; Fig. 2G), which are known to be transported along axons.

### Axonal transport is disrupted in *Elavl3*^**−**/**−**^ Purkinje cells

**Figure 3:**
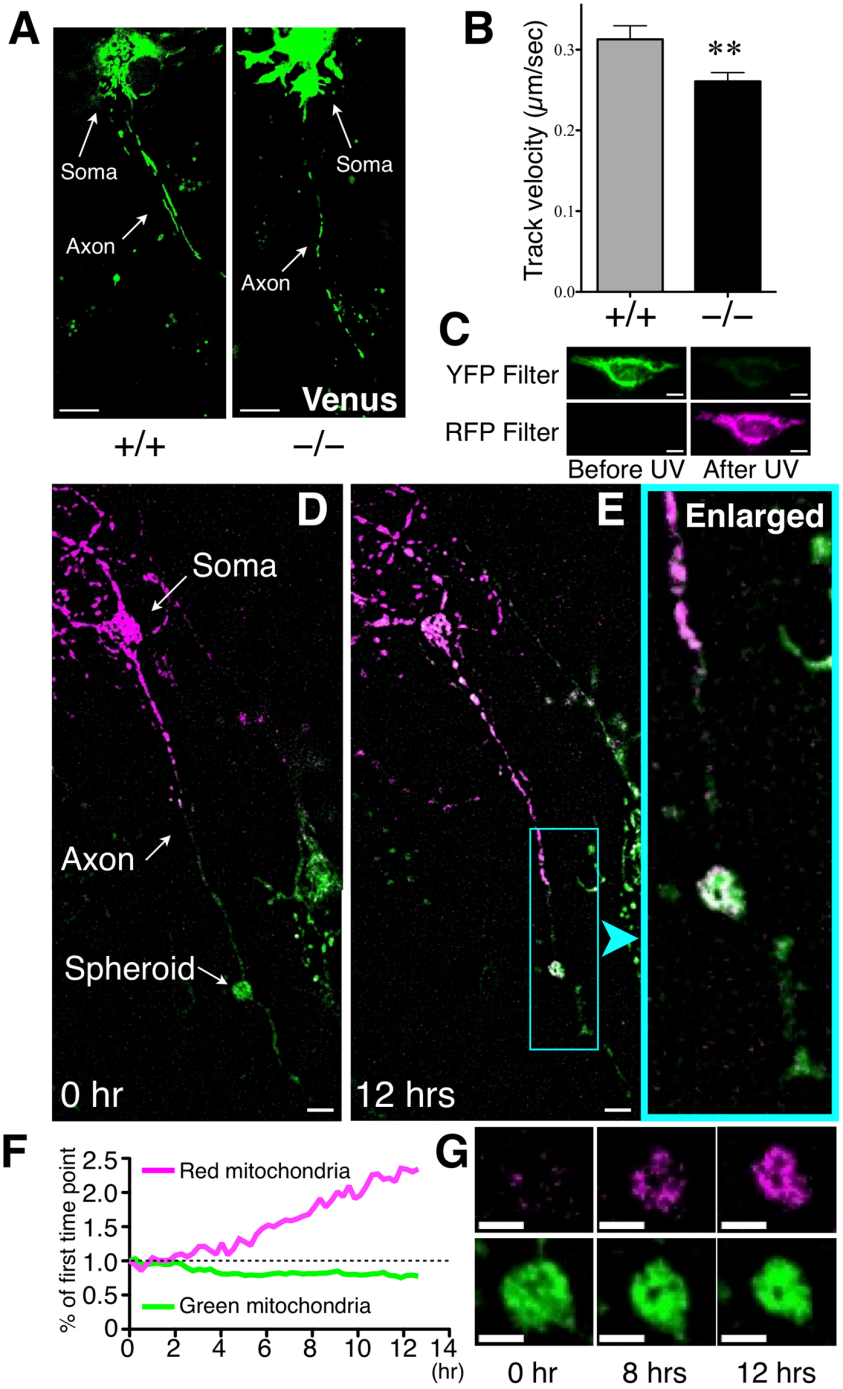
Live imaging analysis of axonal transport. (**A**) Representative images of cultured *Elavl3*^+/+^ or *Elavl3*^−/−^ Purkinje cells expressing mito–Venus under control of the CAG promoter. (**B**) Velocity of mitochondria was semi-automatically calculated using Volocity^®^ software (*Elavl3*^+/+^, n = 160 mitochondria from 9 Purkinje cells; *Elavl3*^−/−^, n = 361 mitochondria from 10 Purkinje cells). **P < 0.01 (Student’s t-test). Scale bar, 10 μm. (**C**) Representative images of HeLa cells expressing mito–KikGR under control of the L7 promoter. The fluorescent color of mito–KikGR is mostly converted from green to red by 60 sec of UV irradiation. Scale bar, 10 μm. (**D–G**) Cultured *Elavl3*^−/−^ Purkinje cells were transfected with a lentiviral vector expressing mito–KikGR under control of the L7 promoter (green) and cytoplasmic mitochondria were photoconverted to red. (**D** and **E**) Representative images of cultured Purkinje cells expressing mito–KikGR at 0 hr (**D**) and at 12 hr (**E**). Scale bar, 10 μm. (**F**) A plot of the mito–KikGR signal intensity within spheroids. The signal intensity of each color of mito–KikGR at the first time point was defined as 1. (**G**) Representative time-course images of spheroid. Red mitochondria from soma gradually accumulated within the spheroid (also see Supplementary movie S3). Scale bar, 5 μm.

Based on the observation that the spheroids were filled with molecules that are transported along axons, dysfunction of axonal transport was suspected in *Elavl3*^−/−^ Purkinje cells. Therefore, we evaluated mitochondrial movement in cultured Purkinje cells using mitochondria-targeted yellow fluorescent protein (mito–Venus) controlled by CAG promoter^29^. Mito–Venus was predominantly expressed in Purkinje cells by *in utero* injection of a replication-defective adenoviral vector (see method), and mitochondrial movement was captured by time-lapse imaging (Fig. 3A). The velocity of mitochondria in *Elavl3*^+/+^ Purkinje cells was 0.32 ± 0.017 µm/sec (mean ± SE) but was significantly decreased in *Elavl3*^−/−^ Purkinje cells to 0.26 ± 0.011 µm/sec (Fig. 3B).

We next evaluated mitochondrial movement within spheroids using the photoconvertible fluorescent protein KikGR; its fluorescent color can be converted from green to red by UV irradiation (Fig. 3C). Cultured Purkinje cells were transfected with lentiviral vectors expressing mitochondria-targeted KikGR (mito–KikGR) under the control of Purkinje cell-specific L7 promoter^30^. The cytoplasmic mitochondria of Purkinje cells, which have apparent axonal spheroids, were photoconverted to red, and time-lapse imaging was then performed (Fig. 3D). Red mitochondria from the soma gradually accumulated within the spheroid but rarely passed beyond the spheroid for 12 hr of observation (Fig. 3E–G**:** Supplementary Movie S3). These results indicate that axonal transport is disturbed by spheroids in *Elavl3*^−/−^ Purkinje cells.

### Downregulation of KIF3A and KIF3C in *Elavl3*^**−**/**−**^ Purkinje cells

**Figure 4:**
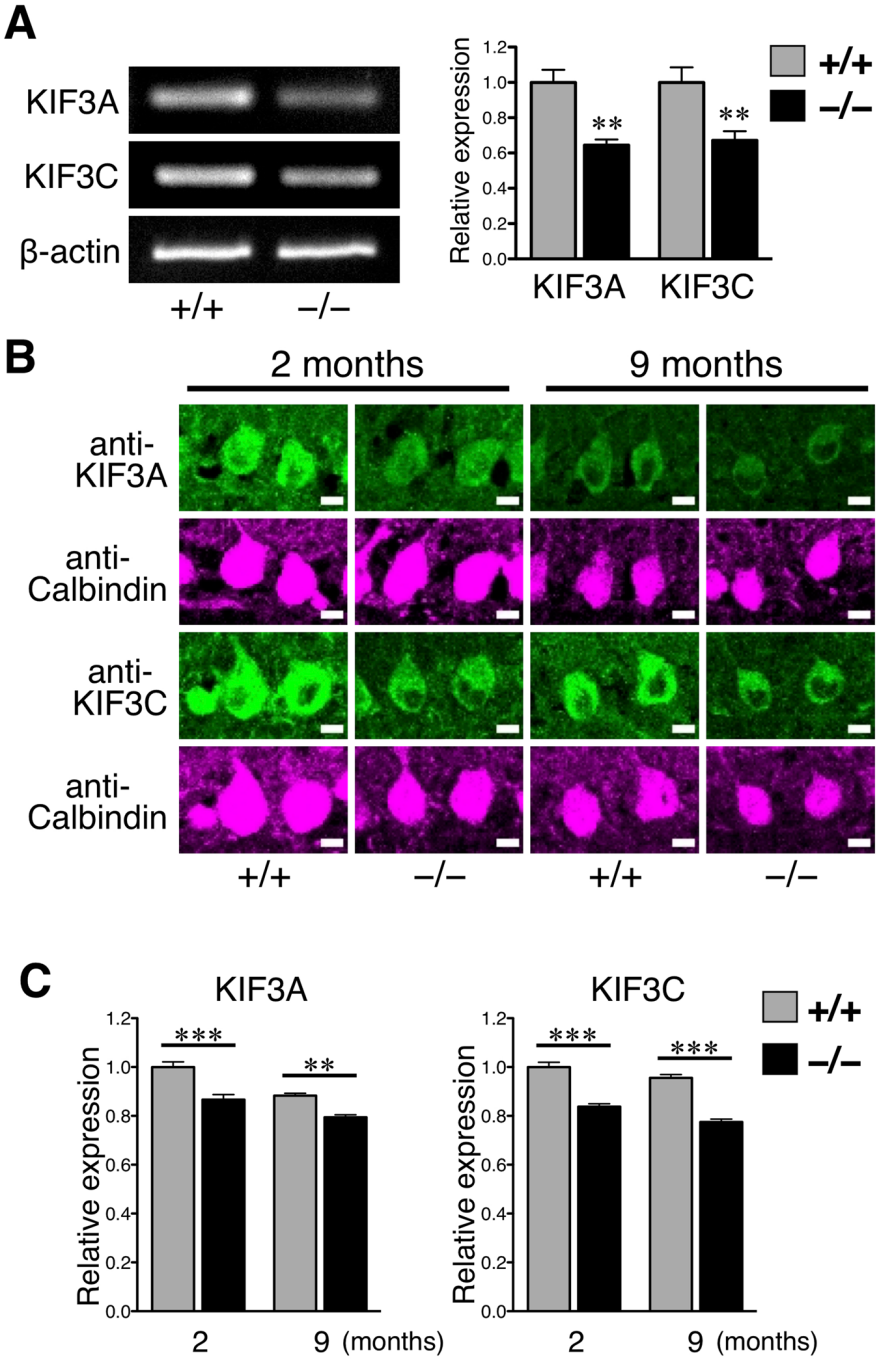
Expression analysis of KIF3A and KIF3C. (**A**) RT-PCR analysis of KIF3A and KIF3C in the cerebella of 2-month-old *Elavl3*^+/+^ and *Elavl3*^−/−^ mice. Expression levels of KIF3A and KIF3C mRNA were significantly decreased in *Elavl3*^−/−^ cerebella. (n = 6 mice for each group). **P < 0.01 (Student’s t-test). Full-length gel is presented in Supplementary Fig. S5. (**B**) The cerebellar sections of *Elavl3*^+/+^ and *Elavl3*^−/−^ mice at 2 or 9 months of age were immunostained with the indicated antibodies. (**C**) The fluorescent signal ratios of KIF3A and KIF3C in the cytosolic region of Purkinje cells were quantified (also see the Methods). The fluorescent signal ratios of KIF3A and KIF3C were significantly decreased in *Elavl3*^−/−^ Purkinje cells in both 2- and 9-month-old mice (n = 316, 288, 298, and 269 Purkinje cells from 2 independent mice for KIF3A, and n = 264, 243, 217, and 262 Purkinje cells from 2 independent mice for KIF3C). Scale bar, 10 μm. *P < 0.05, **P < 0.01, ***P < 0.001 (one-way ANOVA followed by the Bonferroni post hoc test).

Anterograde axonal transport is mainly mediated by the kinesin superfamily (KIF). Mitochondria, lysosomes, endosomes, and synaptic vesicles are well-known cargos of KIF proteins^31,32^. We focused on the kinesin-2 family members KIF3A and KIF3C and evaluated their endogenous expression levels. The mRNA levels of KIF3A and KIF3C were evaluated by semi-quantitative RT-PCR using mRNAs extracted from the whole cerebellum, including granule cells, which are not nElavl null. We found that KIF3A and KIF3C were significantly downregulated in the cerebella of *Elavl3*^−/−^ mice at 2 months of age (Fig. 4A). To determine whether KIF3A and KIF3C are downregulated at the protein level in Purkinje cells, we quantified the signal intensity of KIF3A and KIF3C proteins by immunostaining and specifically considering the region of interest in the cytosol of Purkinje cells (Fig. 4B). We found that KIF3A and KIF3C proteins were significantly downregulated in *Elavl3*^−/−^ Purkinje cells at both 2 and 9 months of age (Fig. 4C).

### Overexpression of KIF proteins enlarged spheroid size

**Figure 5:**
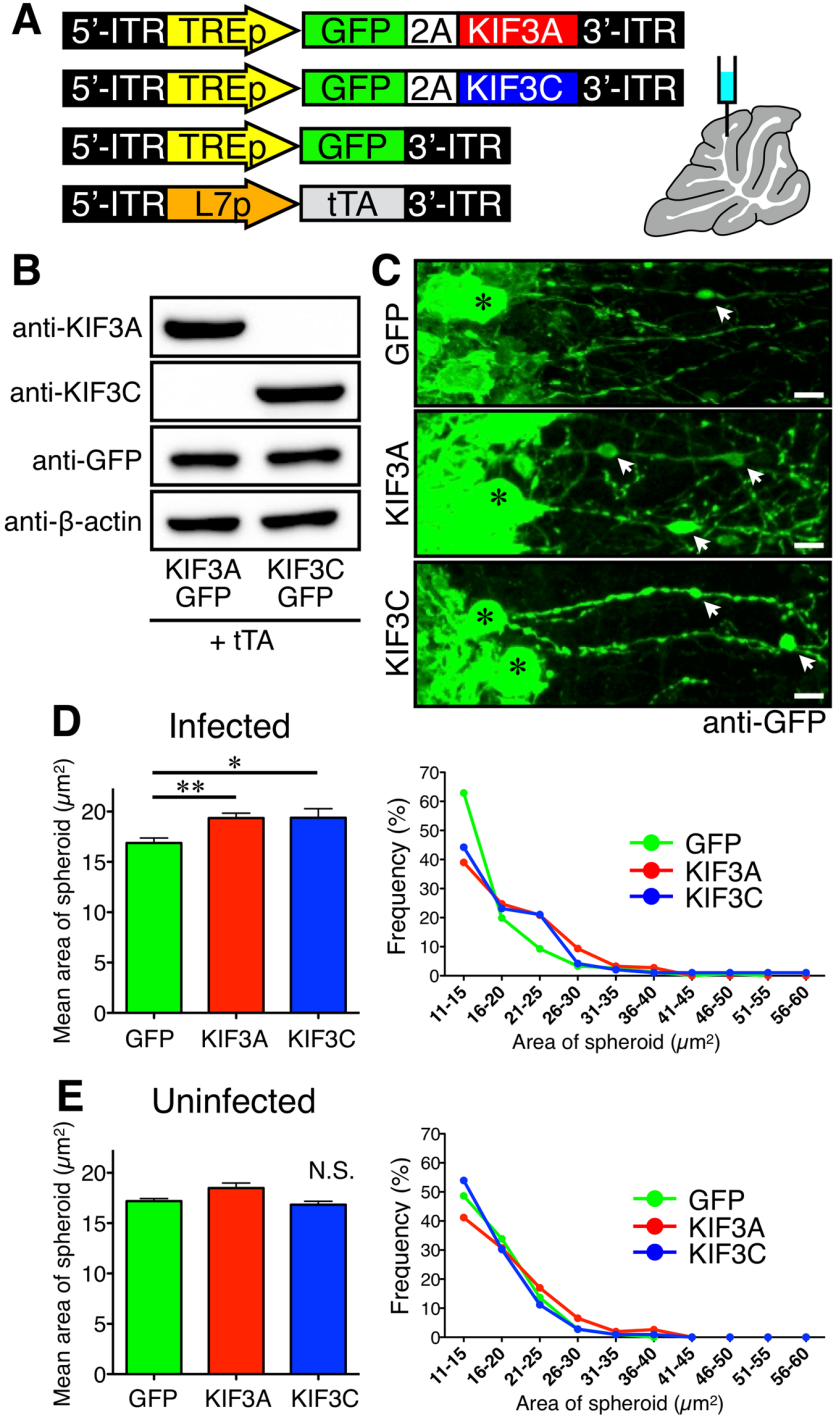
Overexpression of KIF proteins *in vivo*. (**A**) Schematic representation of the expression constructs used in this study, and an illustration of the injection site of the ssAAV9 vector. (**B**) Western blot of HeLa cell lysates co-transfected with constructs expressing tTA under control of the L7 promoter and GFP–2A–KIF3A or GFP–2A–KIF3C under control of the TRE promoter. The expression of KIF3A or KIF3C, together with GFP, was confirmed by the indicated antibodies. Full-length blots are presented in Supplementary Fig. S5. (**C**) Representative images of *Elavl3*^−/−^ Purkinje cells infected with ssAAV9 vectors expressing tTA and GFP, GFP–2A–KIF3A or GFP–2A–KIF3C. Sections were immunostained with anti-GFP antibody. Asterisks and arrows indicate somata of Purkinje cells and axonal spheroids in the granule cell layer, respectively. Scale bar, 10 μm. (**D** and **E**) The areas of each spheroid were measured and plotted in the column graph (left) and the line graph (right). (**D**) The size of spheroids was significantly enlarged by the expression of GFP–2A–KIF3A or GFP–2A–KIF3C compared to controls (GFP) (**D** left; n = 151, 182, and 95 spheroids from 3, 2, and 2 independent mice, respectively). The frequency of the spheroids with sizes of 21 to 25 μm^2^ was increased compared to controls (**D** right). (**E**) The size of spheroids of uninfected Purkinje cells was not changed between groups (n = 292, 153, and 215 spheroids from 3, 2, and 2 independent mice, respectively). *P < 0.05, **P < 0.01 (one-way ANOVA followed by the Bonferroni post hoc test).

To further investigate the association between axonal transport and axonal degeneration, we performed a rescue experiment by overexpressing KIF3A and KIF3C specifically in Purkinje cells of *Elavl3*^−/−^ mice. We conducted gene transfer in Purkinje cells by direct injection of a single-stranded adeno-associated virus serotype-9 (ssAAV9) vector into the cerebellum^33^. To enable sufficient expression of transfected genes, we used a double infection system consisting of tetracycline trans-activator (tTA) under the control of L7 promoter^30^ and GFP, GFP–P2A–KIF3A, or GFP–P2A–KIF3C under the control of tetracycline response element (TRE) promoter, as shown in Fig. 5A. The expression of KIF3A or KIF3C, together with GFP, was confirmed by transfecting HeLa cells with plasmids (Fig. 5B). ssAAV9 vectors were then directly injected into the cerebella of 3-week-old *Elavl3*^+/+^ and *Elavl3*^−/−^ mice. These mice were sacrificed 2–3 months after injection and immunostained with anti-GFP antibody (Fig. 5C). Expression of KIF3A or KIF3C did not prevent spheroid formation. On the contrary, it significantly enlarged the size of the spheroids (Fig. 5D) and, particularly, the frequency of 21- to 25-μm^2^ spheroids was increased compared to control (GFP). However, in each virus-injected animal, the size of spheroids in uninfected Purkinje cells remained unchanged (Fig. 5E). On the other hand, expression of KIF3A or KIF3C did not induce axonal abnormalities for Purkinje cells of *Elavl3*^+/+^ mice (Supplementary Fig. S3).

### Loss of neuronal polarity is suspected in *Elavl3*^**−**/**−**^ Purkinje cells

**Figure 6:**
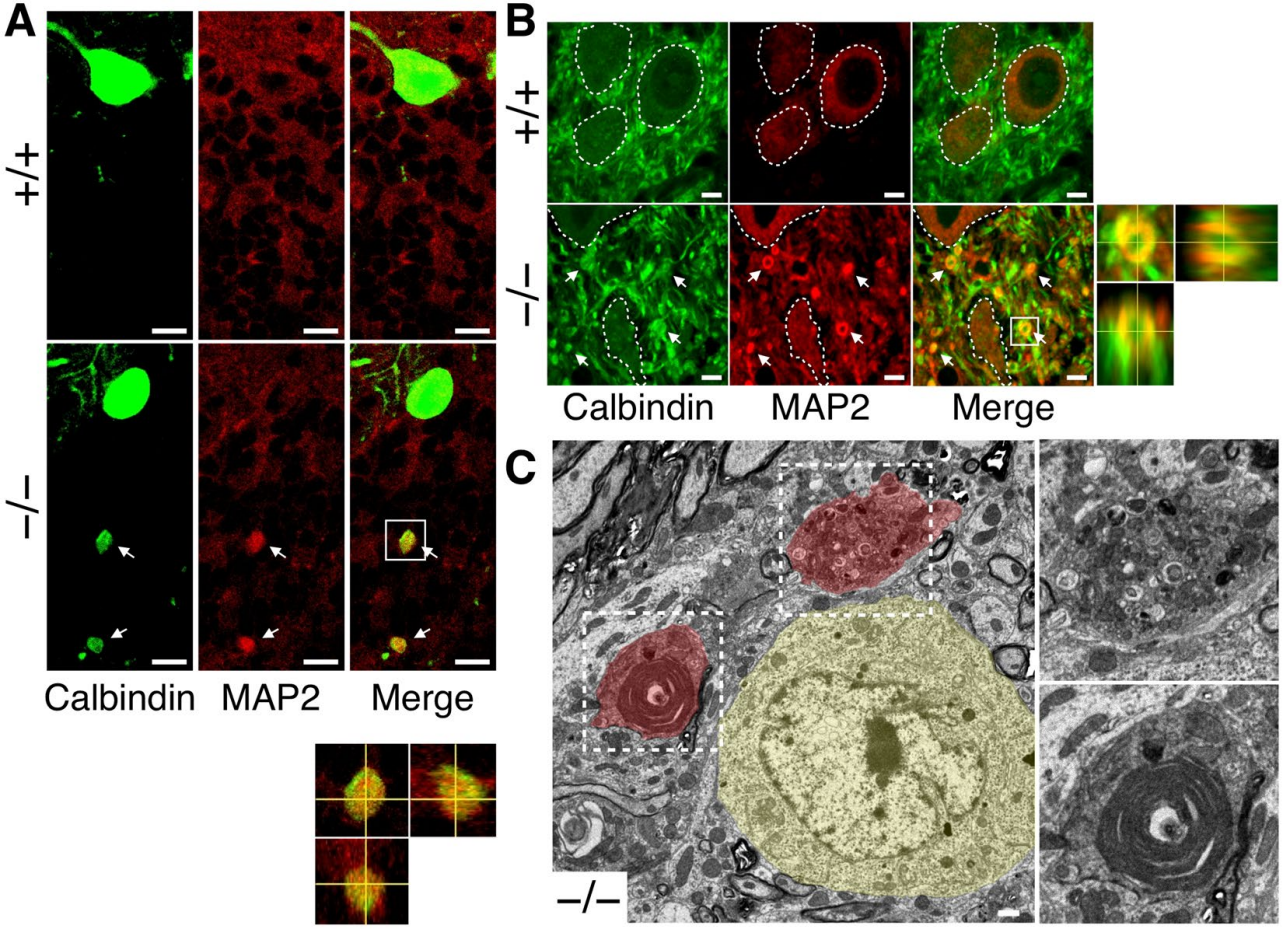
MAP2 protein accumulated within spheroids. (**A** and **B**) The cerebellar sections of *Elavl3*^+/+^ and *Elavl3*^−/−^ mice at 6 months of age were immunostained with anti-MAP2 and calbindin antibodies. Confocal microscopic analysis showed that spheroids in the granule cell layer (**A**) and swollen axonal terminals in the cerebellar nuclei (**B**) were stained with MAP2. Boxed regions in (**A**) and (**B**) are enlarged as orthogonal projections in bottom and right panels, respectively. Somata of cerebellar nuclei neurons in (**B**) are demarcated by a white line. Scale bars: A, 10 μm; B, 5 μm. (**C**) Ultrastructures of cerebellar nuclei of 5-months-old *Elavl3*^−/−^ mice were analyzed via transmission electron microscopy. A cerebellar nuclei neuron is pseudo-colored yellow and abnormal structures are pseudo-colored red. Boxed regions are enlarged in right panels. Scale bar, 1 μm.

We finally found that the spheroids were filled with not only the cargos of axonal transport but also the somato-dendritic protein such as MAP2. Approximately half of the spheroids in the granule cell layer were filled with MAP2 protein (Fig. 6A), and many of the swollen axonal terminals in the cerebellar nuclei were also filled with MAP2 protein (Fig. 6B). Through transmission electron microscopy analysis of cerebellar nuclei, some of the abnormal structures filled with various organelles, which would indicate the MAP2 positive axonal terminals, were observed around the cerebellar nuclei neurons (Fig. 6C).

## Discussion

Young adult *Elavl3*^−/−^ mice exhibit relatively mild motor deficits and seizures, as described previously^17^. One seizure mechanism was explained by glutamate network imbalance induced by insufficient alternative splicing of glutaminase RNA. However, the mechanism of motor deficits is not fully understood, although an association with the complete loss of nElavl proteins in Purkinje cells has been hypothesized.

Here, we show that *Elavl3*^−/−^ mice exhibit slowly progressive motor deficits leading to severe cerebellar ataxia with the progression of axonal degeneration. The degeneration of Purkinje cells is known to cause some types of spinocerebellar ataxia. In these diseases, the cerebellum undergoes loss of Purkinje cells, atrophy and/or disarrangement of anatomical architectures in both clinical patients and animal models^34–37^. On the other hand, although most axons of Purkinje cells are degenerated in aged *Elavl3*^−/−^ mice, somata and dendrites remain mostly intact and appear normal throughout the life span. Intriguingly, a previous study also suggested that the mutations of Elav gene, which induce malformation of neurons, did not induce cell death in *Drosophila*^38^. Then, it is tempting to speculate that the functions of nElavl proteins are indispensable for the long-term maintenance of axonal homeostasis rather than dendrtic and somatic homeostasis in adult brain. Indeed, many targets of nElavl proteins are known to function in the maintenance of axonal structures^17^. Several studies have implicated nElavl genes as candidates linked to neurodegenerative diseases^21–25^, but how the nElavl dysfunction induces or worsens these diseases have not yet been revealed. To our knowledge, this is the first report that demonstrates the direct association between nElavl dysfunction and neuronal degeneration in the adult brain.

A recent study demonstrated that the functions of nElavl proteins are suppressed in the brains of Alzheimer’s disease patients by Y RNAs^26^, a group of noncoding small RNAs. Some Y RNAs contain binding sites for nElavl proteins and prevent nElavl proteins from associating with normal targets as a response to cell stress. Another report also suggested that the expression level of Elavl2 is suppressed in hippocampal neurons by kainic acid stimulation in an activity-dependent manner^39^. These data indicate that the functions of nElavl proteins are suppressed in neurons in response to cellular damage, including exposure to free oxygen radicals or excessive activation, which may accompany microvascular damage or various stresses of daily life. In addition, axonal spheroid formation has also been reported in patients with Parkinson’s disease^40^, Alzheimer’s disease^41,42^, spinocerebellar ataxia^43^, and amyotrophic lateral sclerosis^44^. It is possible that axonal degeneration accompanied by dysfunction of axonal transportation, such as that observed in *Elavl3*^−/−^ mice, also occurs in the human brain and triggers neurodegenerative diseases. This theory supports the previous findings that demonstrated that single-nucleotide polymorphisms in the Elavl4 gene increase the risk of Parkinson’s disease^23–25^.

In this paper, kinesin-2 family members KIF3A and KIF3C are used as representative markers for KIF proteins. We first speculated that downregulation of these KIF proteins is a causal factor of axonal degeneration because KIF3C is reported to promote axonal regeneration^45^ and because KIF3A has been reported to transport N-cadherin and β-catenin^46^ proteins, which are required for synapse formation. Results supporting this theory were not obtained, since spheroid size was enlarged by the expression of these KIF proteins. There is also a possibility that the upregulation of KIF proteins induces spheroid formation. However, it is not likely to be the cause of axonal degeneration, since the overexpression of KIF proteins in *Elavl3*^+/+^ Purkinje cells did not induce axonal abnormalities. Thus, we concluded that the suppression of KIF proteins is not the cause but could be a consequence of axonal degeneration.

Then, what would be the causal factors of axonal degeneration? Previous studies have shown that the spheroids of Purkinje cells appear in several mutant mice (such as “hyperspiny Purkinje cell” mutant mice^47^, cerebroside sulfotransferease (CST) mutant mice^48^, SCA6^84Q/84Q^ knockin mice^49^, and MTCL1 gene trap mice^50^) or as a response to various stresses (such as axotomy^51,52^ and kainic acid-induced lesion^53^). The hypertrophy of recurrent collateral branches of the axons of Purkinje cells is shown to be associated with the formation of spheroids in these findings^49,51–53^. In the present study, we are focusing on the association of the loss of neuronal polarity, which has been reported in the study of MTCL1 deficient mice^50^. MTCL1 is one of the important factors required for the formation of neuronal polarity and the dysfunction of MTCL1 leads the appearance of spheroids in the axons of Purkinje cells. Importantly, previous HITS-CLIP assay^17^ revealed that the nElavl proteins regulate the alternative splicing of some exons in ankyrinG and ankyrinB, critical factors for neuronal polarity formation^54,55^. MAP2 is the well-known somato-dendritic protein that does not normally enter into axons, but leak into axons when neuronal polarity was disrupted^56^. In *Elavl3*^−/−^ Purkinje cells, however, MAP2 accumulated in both the spheroids and swollen axonal terminals. Furthermore, although it was rare, some of the cytosolic organelles, which are normally not detectable in the axons, were observed in the spheroids in our ultrastructural analysis (Y. Ogawa and H. J. Okano, our unpublished observations).

Finally, we propose *Elavl3*^−/−^ mice as an advanced animal model that constantly develops slowly progressive motor deficits leading to severe cerebellar ataxia, similar to human patients. In clinical cases, axonal degeneration is believed to be an early process in the development of neurodegenerative disease that occurs before the onset of signs and symptoms^57,58^. Similar to this concept, axonal degeneration of Purkinje cells was observed much earlier than the onset of severe cerebellar ataxia in *Elavl3*^−/−^ mice, and the formation of spheroids might be related to the relatively milder motor deficits. Moreover, the present study demonstrated how axonal degeneration is associated with subsequent motor dysfunctions. We therefore expect that the mechanisms of and therapeutic approaches for axonal degeneration, which could not be studied by previously developed animal models, will be revealed by studying *Elavl3*^−/−^ mice.

## Materials and Methods

### Animals

This study was approved by the Institutional Animal Care and Use Committee of the Jikei University School of Medicine (approval number: 2015-111C2, 2015-112C4, and 2016-004C2) and the Animal Ethics Committee of Keio University (approval number: 053024). All procedures were performed in full compliance with the Fundamental Guidelines for Proper Conduct of Animal Experiments and Related Activities in Academic Research Institutions issued by the Japanese Ministry of Education, Culture, Sports, Science and Technology. Mice were housed under temperature-controlled conditions (temperature: 24–25 °C) and maintained on a 12-hr light/dark cycle with *ad libitum* access to food and water. *Elavl3*^−/−^ mice were generated as described previously^17^. Mouse lines were maintained on C57BL/6J or ICR backgrounds and genotyped by PCR.

### *In vivo* analysis of motor functions

Mouse gait was analyzed as described in a previous paper^59^ with minor modifications. Briefly, mice were allowed to walk in a custom-built open-top runway (50 cm long, 10 cm wide, with walls 10 cm high) and their movements were captured by video camera from the bottom side. The base width, angle, stance length, stride length, and step cycle of forelimb and hindlimb were quantified manually as shown in Supplementary Fig. S1A. Tremor during walk was scored by two investigators blinded to the experimental design. The score was judged as follows and averaged: 2 = mouse with severe tremor; 1 = mouse with mild tremor or abnormal movement; 0 = mouse with normal behavior.

### Immunohistochemistry and semi-quantification

Mice were deeply anesthetized and fixed by perfusion with 4% paraformaldehyde (PFA) buffered with 0.1 M phosphate buffer. Cerebella were removed and cut into 20- or 40-μm-thick sections using a cryostat (HM525, Thermo Fisher Scientific, Waltham, MA, USA). Sections were immunostained with a mixture of the following antibodies: rabbit polyclonal anti-calbindin D-28K (1:500; AB1778; Chemicon, Billerica, MA, USA), mouse monoclonal anti-calbindin D-28K (1:500; C9848; Sigma-Aldrich, St. Louis, MO, USA), chicken polyclonal anti-MAP2 (1:5000; ab5392; Abcam, Cambridge, UK), guinea pig polyclonal anti-vesicular glutamate transporter 1 (1:100; ab5905; Abcam), mouse monoclonal anti-vesicular glutamate transporter 2 (1:100; ab79157; Abcam), mouse monoclonal anti-amyloid precursor protein (1:100; M009-3; MBL, Nagoya, Japan), mouse monoclonal anti-neurofilament H non-phosphorylated (1:1000; SMI-32R; Covance, Princeton, NJ, USA), rat monoclonal anti-myelin basic protein (1:200; MAB386; Chemicon), mouse monoclonal anti-KIF3A (1:100; ab24626; Abcam), mouse monoclonal anti-KIF3C (1:100; ab89276; Abcam), rabbit polyclonal anti-GFP (1:1000; 598; MBL), and rat monoclonal anti-GFP (1:500; GF090R; Nacalai Tesque, Kyoto, Japan) antibodies. Images were taken with a confocal laser-scanning microscope (LSM 550, 700, and 880, Carl Zeiss, Jena, Germany). For a quantitative analysis of the density of axonal terminals of Purkinje cells at the cerebellar nuclei, the mean signal intensities of calbindin in each image (8000–15000 μm^2^) or the signal intensities of calbindin at the vicinity of each cerebellar nuclei neuron (distance of 10 pixels from the cell membrane) was quantified using ImageJ software (National Institute of Health, Bethesda, MD, USA). Each data was normalized to that of control samples (*Elavl3*^+/+^ and *Elavl3*^+/−^) before 7 months of age. For a semi-quantitative analysis of KIF proteins, the fluorescent intensities of KIF3A or KIF3C in the cytosolic region of Purkinje cells and the fluorescent intensities of the background region were quantified using ImageJ software (National Institute of Health). The fluorescent signal ratios of KIF3A and KIF3C were obtained by dividing the fluorescent intensities of KIF3A and KIF3C by the background intensity. See the Supplementary methods for more details.

### Electron microscopy

Electron microscopy samples were prepared as described previously^60,61^. Ultrathin sections were observed with an electron microscope (H-7100 and HT7700; Hitachi, Tokyo, Japan). See the Supplementary methods for more details.

### Production of recombinant adenoviral vector

Mitochondria-targeted Venus (mito–Venus) was expressed under control of the CAG promoter^29^. An adenoviral vector was generated using pAD/PL–DEST via the Gateway System (Thermo Fisher Scientific). See the Supplementary methods for more details.

### Production of recombinant lentiviral vector

Mitochondria-targeted KikGR (mito–KikGR) was expressed under the control of the Purkinje cell-specific truncated L7 promoter^30^. A lentiviral vector was generated according to the RIKEN BRC lentiviral vector preparation protocol. See the Supplementary methods for more details.

### Production of recombinant single-stranded adeno-associated virus serotype 9 (ssAAV9) vectors

The coding regions of KIF3A, KIF3C and GFP were expressed under the control of tetracycline response element (TRE) promoter. KIF3A and KIF3C were indirectly fused with GFP using the self-cleaving 2A peptide sequence (P2A). To activate the TRE promoter, tetracycline trans-activator (tTA) was expressed under the control of the L7 promoter. Recombinant ssAAV9 vectors were provided by Drs. Ayumu Konno and Hirokazu Hirai. Details of the double infection system will be described in a separate report. See the Supplementary methods for more details.

### Primary culture of Purkinje cells

Brains of E18.5 embryos to P2 pups were dissected in Hank’s balanced salt solution (HBSS) (Thermo Fisher Scientific), and the cerebella were removed and dissociated with 20 mg/ml papain (Sigma-Aldrich). The cells were cultured in poly-lysine (Sigma-Aldrich)-coated glass bottom dishes. The culture medium is described in the Supplementary methods.

### Analysis of mitochondrial movement through axons

For the predominant expression of mito–Venus in Purkinje cells, 1 μl of adenoviral vectors was injected into the ventricles of embryos at embryonic day (E) 11.5 via *in utero* surgery, as described previously^62^. Thereafter, the cerebella of these embryos were dissected and dissociated for culture on E18.5 or later. At 10 days *in vitro* (DIV), mitochondrial movement was recorded by scanning laser confocal microscopy (TCS SP5, Leica Microsystems, Welzlar, Germany). Frames were acquired every 5 sec for a total recording time of 1 min. The trajectory of mitochondrial movement through axons was semi-automatically calculated using Volocity^®^ software (Perkin-Elmer, Boston, MA, USA). The velocity of each mitochondria was calculated from the moved distance and the measurement times. Axons were identified according to morphological criteria, such that the axons of Purkinje cells elongate to long distance without spines.

### Analysis of mitochondrial movement in spheroids

Cultured Purkinje cells were transfected with a lentiviral vector expressing mito–KikGR at 12 DIV. Photoconversion and time-lapse imaging were performed at 18 DIV. Mitochondrial movement was recorded by Deltavision microscopy and processed using SoftWorx^®^ software (Applied Precision, Inc, Issaquah, WA). Frames were acquired every 15 min for a total recording time of 12 hr.

### HeLa cell culture and transfection

HeLa cells were cultured in high glucose DMEM (Sigma-Aldrich) with 10% heat-inactivated fetal bovine serum (Thermo Fisher Scientific) and 100 μg/ml penicillin-streptomycin solution. Expression plasmids were transfected by Lipofectamine^®^ 3000 (Invitrogen, Carlsbad, CA, USA), according to the manufacturer’s protocol.

### RNA isolations and RT-PCR

Total RNA was extracted from mouse brains using TRIzol^®^ reagent (Invitrogen) and an RNeasy^®^ Plus Mini Kit (Qiagen, Hilden, Germany). First-strand cDNAs were synthesized using ReverTra Ace (TOYOBO, Osaka, Japan), according to the manufacturer’s protocol. PCR amplification was performed by KOD -plus- enzyme (TOYOBO). Amplified PCR products were electrophoresed on agarose gels and visualized by staining with ethidium bromide. Primer sets are described in the Supplementary methods.

### Protein extraction and western blotting

Protein extraction from cultured HeLa cells was performed using NP40-based buffer and a protease inhibitor cocktail (Complete Mini; Roche Diagnostics, Rotkreuz, Switzerland). Cells were incubated for 15 min on ice, followed by centrifugation at 15,000 rpm for 15 min. The supernatants were collected as protein samples. Samples were separated using SDS-PAGE and transferred to polyvinylidene difluoride (PVDF) membranes (Millipore, Darmstadt, Germany). Membranes were immunoblotted with following antibodies: mouse monoclonal anti-KIF3A (1:2,000; ab24626; Abcam), mouse monoclonal anti-KIF3C (1:2,000; ab89276; Abcam), rabbit polyclonal anti-GFP (1:10,000; 598; MBL), and mouse monoclonal anti-β-actin (1:10,000; A1978; Sigma-Aldrich). See the Supplementary methods for more details.

### Viral infection and spheroid size analysis

The ssAAV9 vectors were directly injected into the cerebella of 3-week-old mice as described previously^63^. Eight microliters of ssAAV9 vectors were injected at a rate of 267 nl/min for 30 min using a microprocessor-based controller (Narishige, Tokyo, Japan). The titer of each virus is given below. For GFP expression, a mixture of ssAAV9 vectors expressing tTA (titer: 0.2–1 × 10^10^ vg) and GFP (titer: 0.2–1 × 10^10^ vg) was injected. For KIF3A or KIF3C expression, a mixture of ssAAV9 vectors expressing tTA (titer: 1–2 × 10^10^ vg) and GFP–P2A–KIF3A or GFP–P2A–KIF3C (titer: 2–4 × 10^10^ vg) was injected. Two to three months after injection, these mice were sacrificed, and the cerebellar sections were immunostained for GFP and calbindin using a free-floating protocol. Images of Purkinje cells were taken, and the areas of each spheroid were measured using ImageJ software (National Institute of Health). For objective observation, axonal swelling with an area of 11 μm^2^ or more was defined as a spheroid.

### *In vivo* magnetic resonance imaging (MRI)

*Elavl3*^+/+^ and *Elavl3*^−/−^ mice at 8 months of age were anesthetized using isoflurane. MRI scans were obtained on a 9.4 T Biospec 94/20 MRI (Bruker BioSpin; Ettlingen, Germany) with the Cryogenic 2ch surface probe coil (Bruker BioSpin; Ettlingen, Germany) with actively shielded gradients at a maximum strength of 300 mT/m. T2-weighted images were acquired using an echo-planner acquisition spin-echo sequence with the following parameters: time repetition (TR)/time echo (TE): 9000 msec/25.0 msec, field of view (FOV): 23.0 mm × 15.4 mm, matrix: 192 × 128, phase encode segments: 16, slice thickness: 0.3 mm and total scan time: 144 sec. The volume of cerebella and whole brain was measured using BrainSuite and ITK-SNAP software.

### Statistical analysis

Data are expressed as the mean and SEM. Statistical analysis was performed using GraphPad PRISM software (GraphPad Software, La Jolla, CA, USA). The Student’s t-test was used to compare two groups, and one-way analysis of variance (ANOVA) with the Bonferroni post hoc test was used to compare more than two groups.

## Electronic supplementary material

Supplementary information

Supplementary Movie S1

Supplementary Movie S2

Supplementary Movie S3
