# Supplementary material for: Elavl3 is essential for the maintenance of Purkinje neuron axons
Source: Sci Rep. 2018 Feb 9;8:2722. doi: 10.1038/s41598-018-21130-5 (PMC5807307; doi:10.1038/s41598-018-21130-5)
Supplement: Supplementary file 1 — Supplementary information [file 41598_2018_21130_MOESM1_ESM.pdf]

Elavl3 is essential for the maintenance of Purkinje neuron axons

Yuki Ogawa[1,10], Kyoko Kakumoto[2,3,10], Tetsu Yoshida[2,4], Ken-ichiro Kuwako[2], Taisuke Miyazaki[5], Junji Yamaguchi[6], Ayumu Konno[7], Junichi Hata[2,4], Yasuo Uchiyama[6], Hirokazu Hirai[7,8], Masahiko Watanabe[5], Robert B. Darnell[9], Hideyuki Okano[2,4], Hirotaka James Okano[1,2]

[1]Division of Regenerative Medicine, The Jikei University School of Medicine, 3-25-8 Nishi-Shimbashi, Minato-ku, Tokyo 105-8461, Japan.
[2]Department of Physiology, Keio University School of Medicine, 35 Shinanomachi, Shinjuku-ku, Tokyo 160-8582, Japan.
[3]Current Address is Immunoregulation for the treatment of inflammation-related disorders, IBRI Laboratory, Foundation for Biomedical Research and Innovation, 2-2 Minatojima-minamimachi Chuo-ku, Kobe 650-0047, Japan
[4]Laboratory for Marmoset Neural Architecture, Brain Science Institute RIKEN, 2-1 Hirosawa, Wako, Saitama 351-0198, Japan.
[5]Department of Anatomy, Hokkaido University Graduate School of Medicine, Kita 15, Nishi 7, Kita-ku, Sapporo 060-8638, Japan
[6]Department of Cellular and Molecular Neuropathology, Juntendo University Graduate School of Medicine, 2-1-1 Hongo, Bunkyo-ku, Tokyo 113-8421, Japan.
[7]Department of Neurophysiology & Neural Repair, Gunma University Graduate School of Medicine, 3-39-22 Showa-machi, Maebashi, Gunma 371-8511, Japan.
[8]Research Program for Neural Signaling, Division of Endocrinology, Metabolism and Signal research, Gunma University Initiative for Advanced Research, 3-39-22 Showa-machi, Maebashi, Gunma 371-8511, Japan.
[9]Laboratory of Molecular Neuro-Oncology and Howard Hughes Medical Institute, The Rockefeller University, 1230 York Avenue, New York, NY 10065, USA
[10]These authors contributed equally to this work.

Supplementary materials and methods

Immunohistochemistry. Mice were deeply anesthetized and tissues were fixed by perfusion with 4% PFA buffered with 0.1 M phosphate buffer. Brains were removed and postfixed in 4% PFA at 4 °C overnight. After that, cerebella were cryoprotected with 10%, 20%, and 30% sucrose solutions, embedded in OCT compound (Sakura Finetek, Tokyo, Japan), and quickly frozen. The cerebella were cut into 20- or 40-μm-thick sections using a cryostat (HM525, Thermo Fisher Scientific). Sections (20 μm thick) were placed on MAS-coated slides and immunostained, and 40-μm-thick sections were immunostained by a free-floating protocol. The 20-μm-thick sections placed on slides were incubated for 60 min at room temperature (RT) with blocking solution containing 5% bovine serum albumin (BSA) in PBS. Thereafter, the sections were incubated overnight at 4 °C with a mixture of primary antibodies. The antibodies were then rinsed three times with PBS and incubated with the appropriate secondary antibodies conjugated with Alexa™ 488, 546 or 647 (1:400; Molecular Probes, OR, USA) for 2 hr at RT. When necessary, sections were autoclaved in 10 mM citrate buffer (pH 6.0) at 105 °C for 10 min for antigen retrieval before the blocking step. For 40-μm-thick sections, a free-floating protocol was used. In this case, according to the above protocol for 20-μm-thick sections, 0.1% TritonX-100 was added to each blocking and antibody solutions, and all procedures were performed at RT.

Electron microscopy. Mice were fixed with 2% paraformaldehyde and 2% glutaraldehyde buffered with 0.1 M sodium cacodylate buffer. The brains were removed, and the cerebella were postfixed with 2% OsO_4 with 0.1 M sodium cacodylate buffer, block-stained in 1% uranyl acetate, dehydrated with a graded series of alcohol, and embedded in Epon 812 (TAAB, Reading, UK). Ultrathin sections were cut with a UCT ultramicrotome (Leica Microsystems), stained with uranyl acetate and lead citrate and observed with an electron microscope (H-7100 and HT7700; Hitachi).

Production of recombinant adenoviral vector. An adenoviral vector expressing Mito–Venus was constructed as below. Venus with a mitochondria-targeted signal was a gift from Dr. Hiroyuki Miyoshi (RIKEN BRC; taken from lentiviral vector CSII–CMV–Venus–mito–IRES2–Bsd). The DNA fragment of mito–Venus was subcloned into pCAGGS vectors[1], which allowed the expression of mito–Venus under control of the CAG promoter. An adenoviral vector was generated using pAD/PL–DEST via the Gateway System (Thermo Fisher Scientific).

Production of recombinant lentiviral vector. A lentiviral vector expressing mito–KikGR was constructed as below. Photoconvertible fluorescent protein KikGR was purchased by MBL (Nagoya, Japan), and the mitochondria-targeted signal COX VIII was conjugated by PCR. The DNA fragment of mito–KikGR was subcloned into CS–CDF–CG–PRE vectors (kindly gifted by Dr. Hiroyuki Miyoshi, RIKEN BRC, Ibaraki, Japan) instead of the GFP region. For Purkinje cell-specific gene expression, the CMV promoter region was exchanged with the Purkinje cell-specific L7 promoter. Lentiviral vectors were generated according to the RIKEN BRC lentiviral vector preparation protocol.

Production of recombinant ssAAV9 vectors. The coding regions of KIF3A (NM_008443.4) and KIF3C (NM_008445.2) were cloned from a cDNA library derived from mouse brain extract. Each obtained clone was inserted into the expression plasmid pAAV/TREp–GFP–P2A in-frame. Recombinant ssAAV9 vectors were produced by transfection of HEK293T cells with pAAV2/9 (kindly provided by Dr. J. Wilson), a helper plasmid (Stratagene, La Jolla, CA, USA) and pAAV/TREp–GFP–P2A–KIF3A, pAAV/TREp–GFP–P2A–KIF3C, or pAAV/TREp–GFP expression plasmids, as previously described[2]. To activate the TRE promoter specifically in Purkinje cells, ssAAV9 vectors expressing tTA under control of the L7 promoter were similarly produced using the

pAAV/L7p–tTA expression plasmid. The tTA-expressing vectors were mixed with one of the vectors carrying the TRE promoter before injection.

Culture medium of Purkinje cells. DMEM/F12 (Sigma-Aldrich) supplemented with 1% AlbuMax (Thermo Fisher Scientific), B27 supplement (Life Technologies, Rockville, MD, USA), 5% heat-inactivated fetal bovine serum, 2 mM glutamine, 0.6% glucose, 1.125 mg/ml $NaHCO_3$, 0.05 M HEPES, 23 mg/ml insulin, 93 mg/ml transferrin, 28 nM sodium selenite, 19 nM progesterone, 9 mg/ml putrescine, and 10 nM thyroid hormone (L-3,3',5-triiodothyronine).

Primer sets used in RT-PCR. KIF3A (5'-catgcacgtcaggatgggc; 5'-cgggtcagtttggagttccg), KIF3C (5'-catgcggattgaagagaggt; 5'-gcagcctcctccttcctact) and β-actin (5'-gcaccacaccttctacaatg; 5'-tgcttgctgatccacatctg).

Protein extraction and western blotting. Protein extraction from cultured HeLa cells was performed using NP40-based buffer containing 1% NP40, 50 mM Tris (pH 8.0), 150 mM NaCl, and protease inhibitor cocktail (Complete Mini; Roche Diagnostics). This buffer was added to each culture well. Cells were incubated for 15 min on ice, followed by centrifugation at 15,000 rpm for 15 min. The supernatants were collected as protein samples. Protein samples were diluted with an equal volume of 2 × Laemmli sample buffer (Bio Rad, Hercules, CA, USA) supplemented with 5% 2-mercaptoethanol and then incubated for 5 min at 95 °C. Samples were separated using SDS-PAGE and transferred to PVDF membranes (Millipore). Membranes were blocked with 5% non-fat skim milk for 60 min at RT and then incubated overnight at 4 °C with the mixture of primary antibodies. The antibodies were then rinsed three times with TBS containing 0.1% Tween 20 and incubated with an appropriate horseradish peroxidase (HRP)-conjugated secondary antibody (1:5,000; Millipore) for 1 hr at RT. Thereafter, signals were detected using Chemiluminescence HRP Substrate (Takara Bio, Shiga, Japan) with a Luminograph I (ATTO, Tokyo, Japan).

References

1. Niwa, H., Yamamura, K. & Miyazaki, J. Efficient selection for high-expression transfectants with a novel eukaryotic vector. *Gene* **108,** 193–199 (1991).
2. Konno, A. *et al.* Mutant ataxin-3 with an abnormally expanded polyglutamine chain disrupts dendritic development and metabotropic glutamate receptor signaling in mouse cerebellar Purkinje cells. *Cerebellum* **13,** 29–41 (2014).

Supplementary Figure S1.

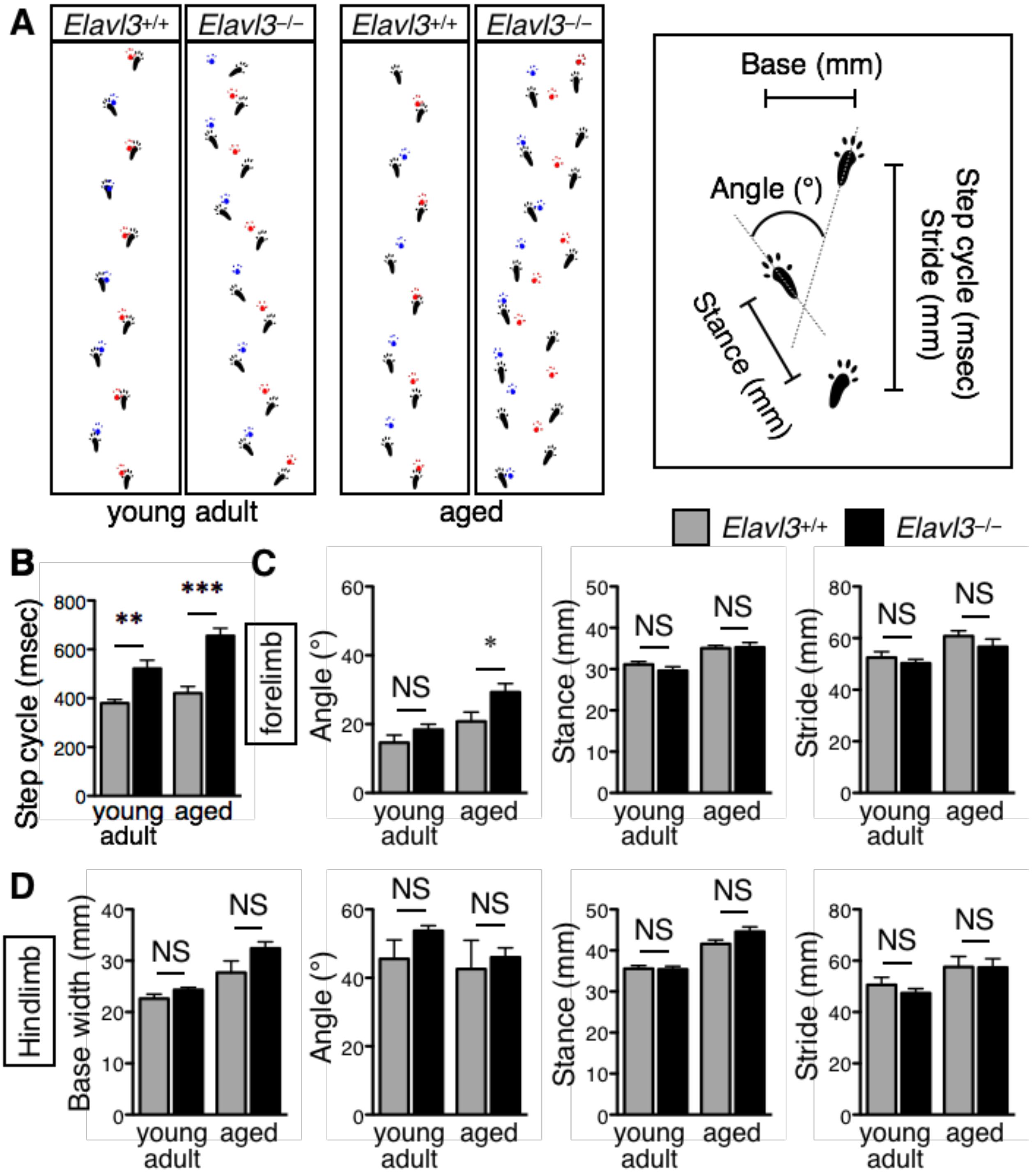

Supplementary Figure S1. Behavioral analysis of *Elavl3*$^{-/-}$ mice.
(A) Representative footprints of forelimb and hindlimb of *Elavl3*$^{+/+}$ and *Elavl3*$^{-/-}$ mice. The left forelimb is colored blue, right forelimb is colored red, and left and right hindlimbs are colored black. Young adult: 2–3 months old; aged: 6–7 months old. The right panel indicate the parameters measured in this analysis. **(B–D)** Comparison of each parameter such as step cycle, angle, stance, stride, and base width measured by forelimb **(C)** or hindlimb **(B and D)** (n = 7, 7, 6, and 7 mice for each group). *P < 0.05, **P < 0.01, ***P < 0.001, NS, not significant (Student's t-test).

Supplementary Figure S2.

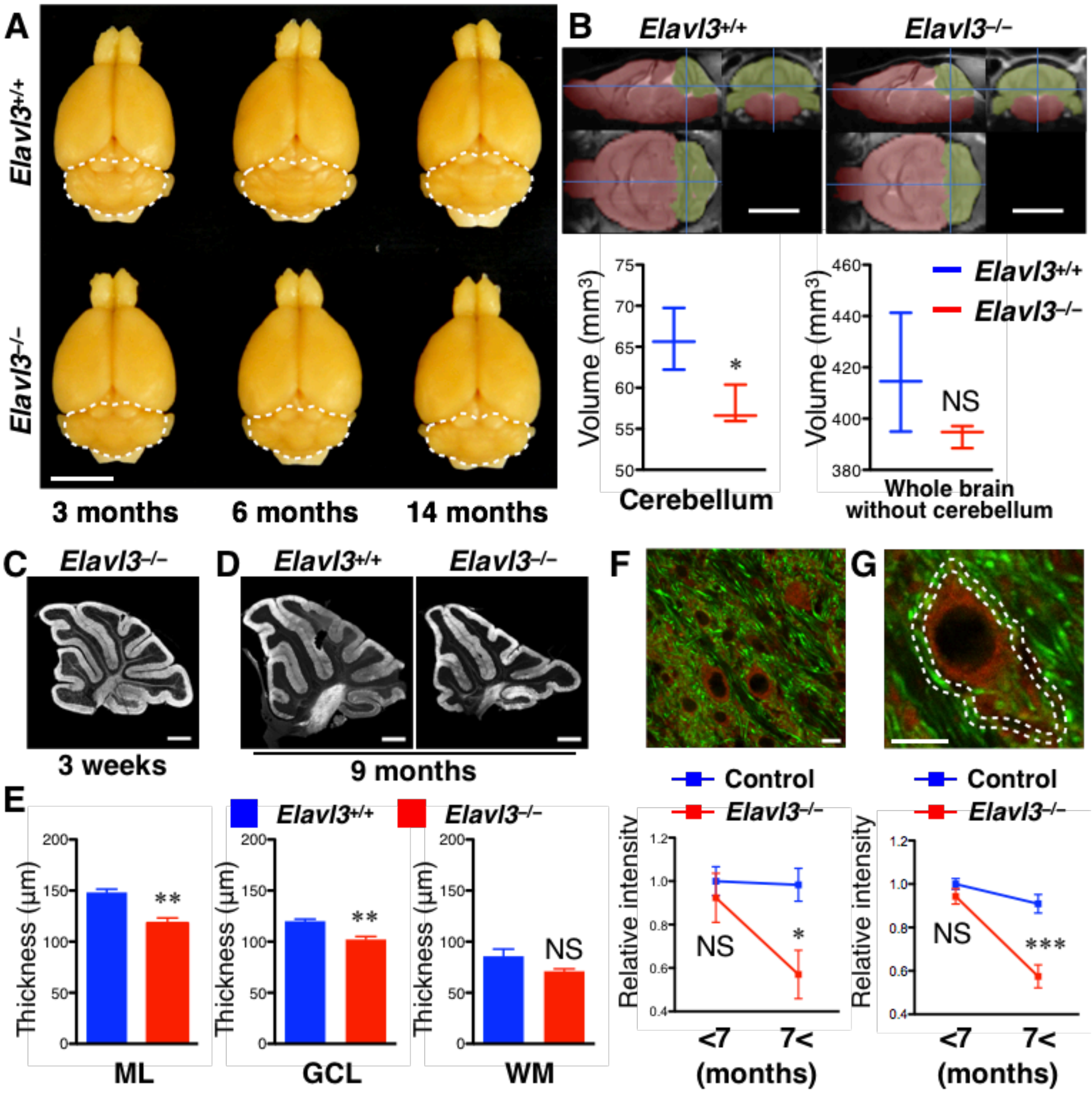

Supplementary Figure S2. Morphological analysis of *Elavl3*$^{-/-}$ cerebella.

(A) The cerebella of *Elavl3*$^{-/-}$ mice appeared slightly smaller than the cerebella of *Elavl3*$^{+/+}$ mice, but the gross anatomy of the cerebella was not markedly different between the *Elavl3*$^{+/+}$ and *Elavl3*$^{-/-}$ mice. No obvious changes were observed in the anatomical architecture of the *Elavl3*$^{-/-}$ cerebellum with aging. Scale bar, 5 mm. **(B)** The brain volume was quantified by *in vivo* magnetic resonance imaging (MRI). The upper panels show the representative MRI of *Elavl3*$^{+/+}$ and *Elavl3*$^{-/-}$ mice at 8 months of age. Cerebellum is colored green, and whole brain without cerebellum is colored red. In *Elavl3*$^{-/-}$ mice, the volume of cerebella was significantly smaller, and the volume of whole brains without cerebella was not significantly different. n = 3 mice for each group. Scale bar, 5 mm. *P < 0.05, NS, not significant (Student's t-test). **(C)** A representative image of cerebellar section of *Elavl3*$^{-/-}$ mice at 3 weeks of age immunostained for calbindin. Scale bar, 500 µm. **(D)** Representative images of cerebellar sections of *Elavl3*$^{+/+}$ and *Elavl3*$^{-/-}$ mice at 9 months of age immunostained for calbindin. Scale bar, 500 µm. **(E)** In *Elavl3*$^{-/-}$ mice, the thickness of molecular layer (ML) and granule cell layer (GCL) was significantly thinner and that of white matter (WM) was not significantly different. n = 4 slices from 4 independent mice at 3–14 months of age for each group. **P < 0.01, NS, not significant (Student's t-test). **(F and G)** The density of axonal terminals of Purkinje cells at the cerebellar nuclei was quantified by two methods. **(F)** The mean signal intensities of calbindin at the cerebellar nuclei of *Elavl3*$^{-/-}$ mice was significantly lower after 7 months of age compared to that of control (*Elavl3*$^{+/+}$ and *Elavl3*$^{+/-}$). Control (<7), n = 6 slices from 5 independent mice; control (7<), n = 3 slices from 3 independent mice; *Elavl3*$^{-/-}$ (<7), n = 5 slices from 5 independent mice; *Elavl3*$^{-/-}$ (7<), n = 5 slices from 3 independent mice. Scale bar, 10 µm. *P < 0.05, NS, not significant (Student's t-test). **(G)** The signal intensities of calbindin at the vicinity of each cerebellar nuclei neuron (distance of 10 pixels from the cell membrane: area between dotted lines) of *Elavl3*$^{-/-}$ mice was significantly lower after 7 months of age compared to that of control (*Elavl3*$^{+/+}$ and *Elavl3*$^{+/-}$). Control (<7), n = 31 Purkinje cells from 5 independent mice; control (7<), n = 17 Purkinje cells from 3 independent mice; *Elavl3*$^{-/-}$ (<7), n = 29 Purkinje cells from 5 independent mice; *Elavl3*$^{-/-}$ (7<), n = 33 Purkinje cells from 3 independent mice. Scale bar, 10 µm. ***P < 0.001, NS, not significant (Student's t-test).

Supplementary Figure S3.

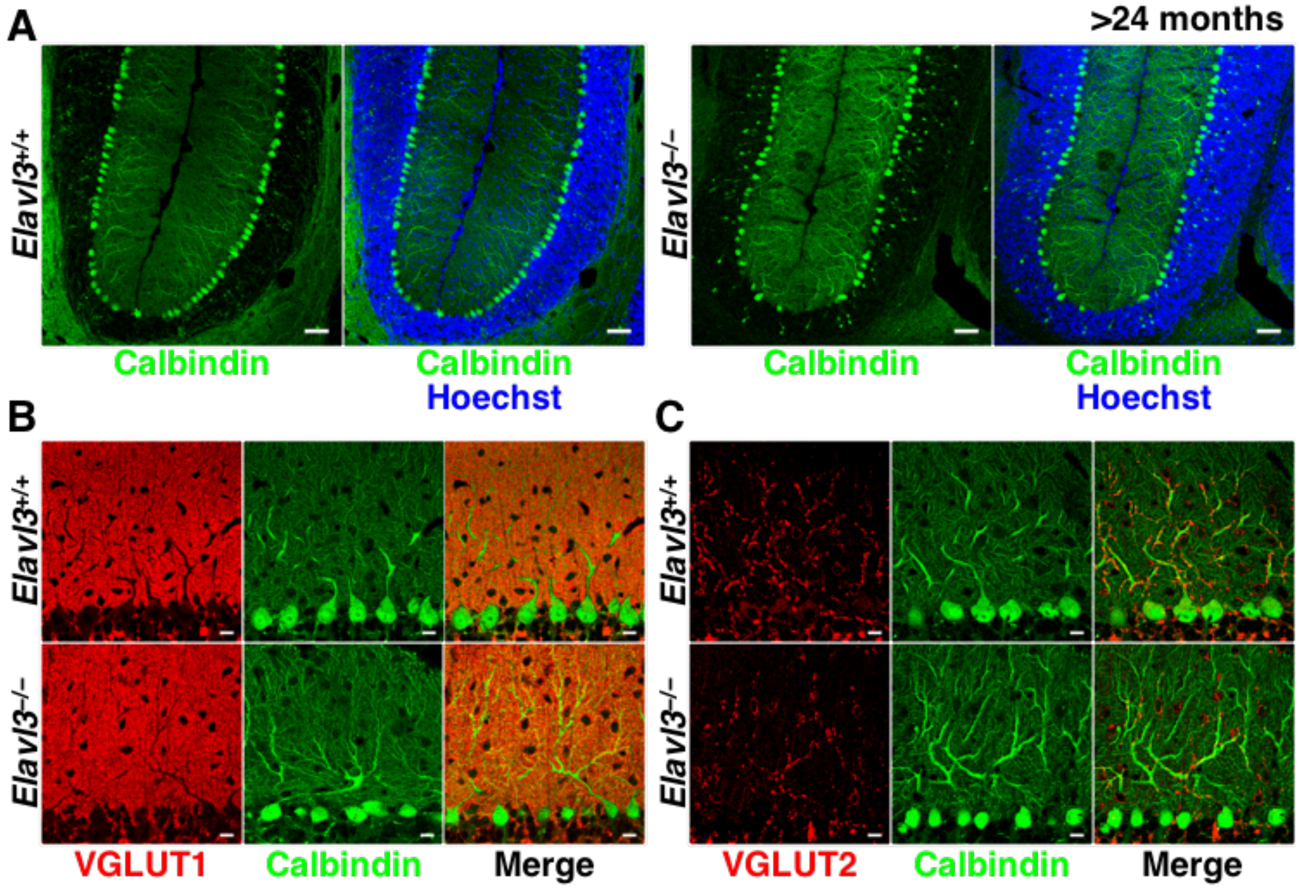

Supplementary Figure S3. Morphological analysis of Purkinje cells in aged *Elavl3*$^{-/-}$ mice.
(A) Somata and dendrites of Purkinje cells were mostly intact, and the laminar structures of cerebellum were retained in the *Elavl3*$^{-/-}$ mice older than 24 months of age. Scale bar, 50 µm. **(B and C)** Synaptic formation of dendrites of Purkinje cells with VGLUT1 positive parallel fibers **(B)** and with VGLUT2 positive climbing fibers **(C)** were observed in the *Elavl3*$^{-/-}$ mice older than 24 months of age. Note that the number of VGLUT2 positive puncta decreased approximately to 60% in *Elavl3*$^{-/-}$ mice. Scale bar, 10 µm.

Supplementary Figure S4.

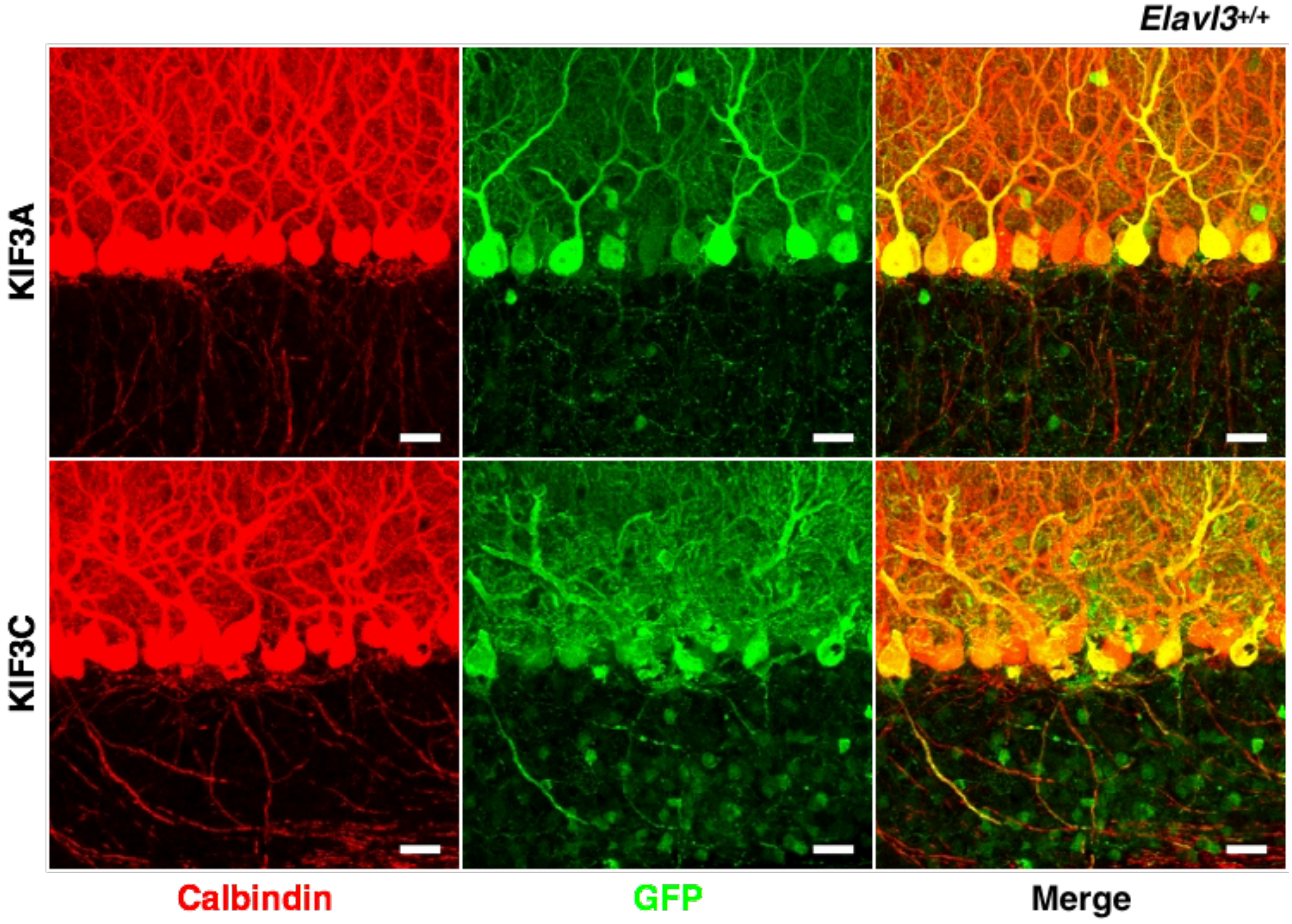

Supplementary Figure S4. Overexpression of KIF proteins to *Elavl3*$^{+/+}$ mice *in vivo*.
Purkinje cells of *Elavl3*$^{+/+}$ mice overexpressing KIF3A or KIF3C did not exhibit axonal abnormalities. Scale bar, 20 μm.

Supplementary Figure S5.

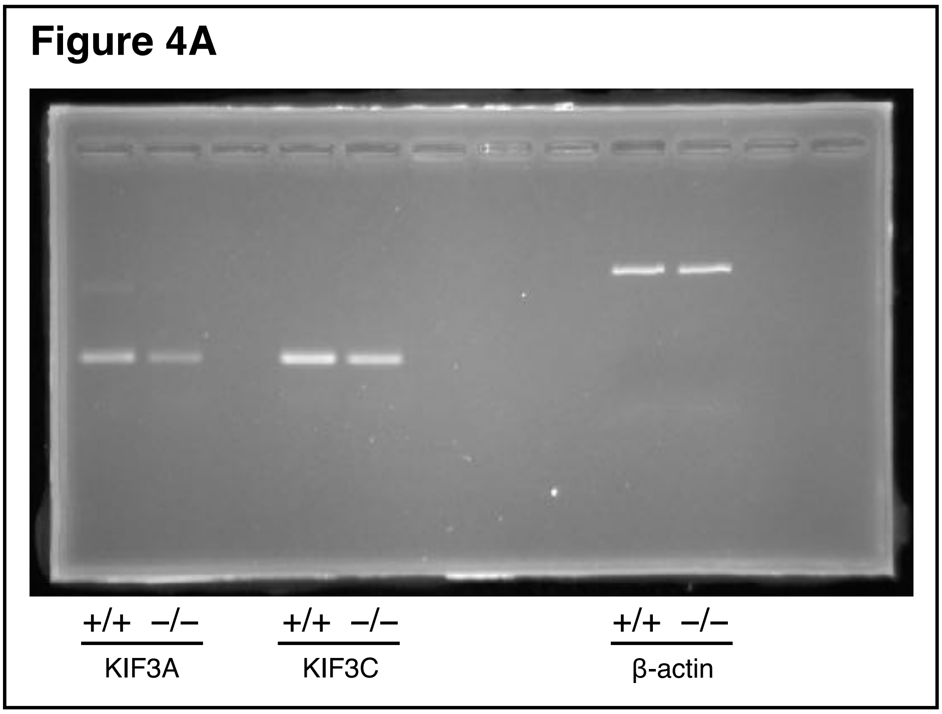

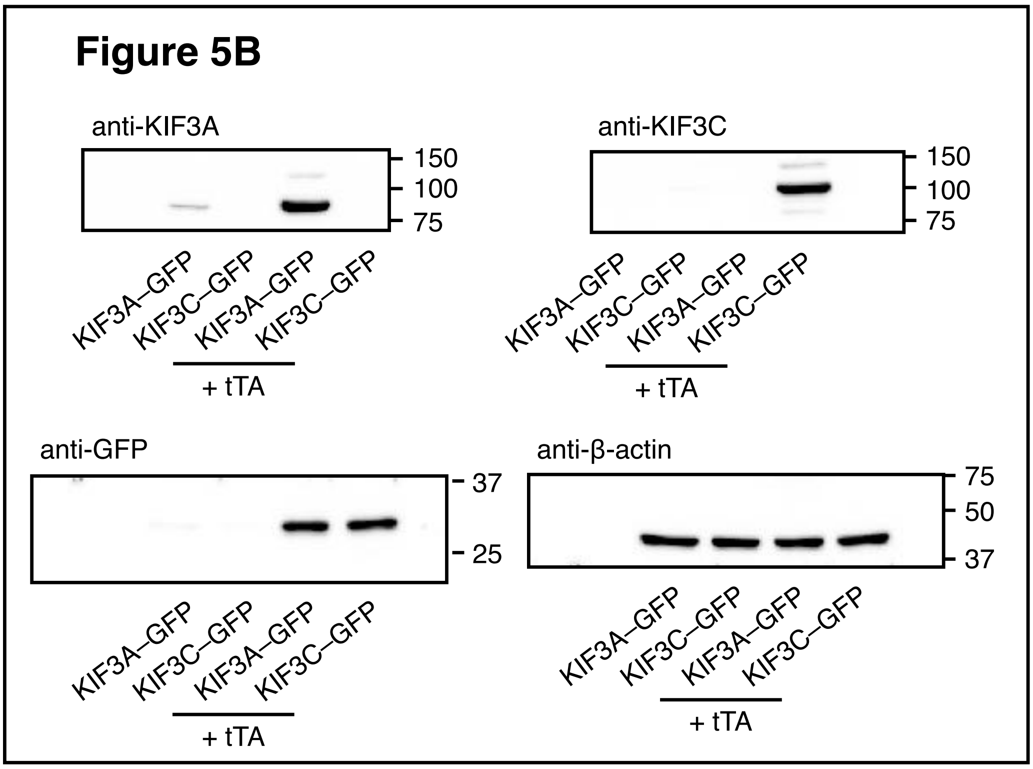

Supplementary Figure S5. Full-length gel and blots presented in main figures.

Supplementary Movie S1. Gait analysis of *Elavl3*$^{-/-}$ mice. Representative video of mouse gait test. Two- and seven-months-old *Elavl3*$^{-/-}$ mice walked with wider base, and 7-months-old *Elavl3*$^{-/-}$ mice also exhibited apparent tremor.

Supplementary Movie S2. Behavioral analysis of *Elavl3*$^{-/-}$ mouse. Thirteen-month-old *Elavl3*$^{-/-}$ mouse exhibited gait abnormalities and postural reflex impairment.

Supplementary Movie S3. Time-lapse imaging of mito–KikGR in the spheroid. Cultured *Elavl3*$^{-/-}$ Purkinje cells were transfected with a lentiviral vector expressing mito–KikGR (green) under control of the L7 promoter, and cytoplasmic mitochondria were photoconverted to red. Red mitochondria from soma gradually accumulated within the spheroid but rarely passed beyond the spheroid in 12 hr of observation. Time is given as hr:min. Scale bar, 10 μm.
